# Illumina sequencing data of the complete chloroplast genome of rare species *Juniperus seravschanica* (Cupressaceae) from Kazakhstan

**DOI:** 10.1016/j.dib.2022.108866

**Published:** 2022-12-29

**Authors:** Moldir Yermagambetova, Saule Abugalieva, Yerlan Turuspekov, Shyryn Almerekova

**Affiliations:** a Molecular Genetics Laboratory, Institute of Plant Biology and Biotechnology, Almaty, Kazakhstan; b Faculty of Biology and Biotechnology, Al-Farabi Kazakh National University, Almaty, Kazakhstan

**Keywords:** Cupressaceae, *Juniperus seravschanica*, Rare species, Illumina sequencing, Chloroplast genome, De novo assembly, Chloroplast SSRs

## Abstract

The species of the genus *Juniperus* L. play an important role in Kazakhstan forest ecosystems and one of them is *Juniperus seravschanica* Kom. which has been listed as a rare species in the Red Book of Kazakhstan. The distribution area of *J. seravschanica* extends from Central Asia (Kazakhstan, Uzbekistan, Kyrgyzstan, Tajikistan, and Turkmenistan) to northern and eastern Afghanistan, northern Pakistan, Kashmir, southeastern Iran, and Oman. *J. seravschanica* occurred in the southern part of Kazakhstan along with the ranges Karatau, Talas Alatau, Kyrgyz Alatau, Chu-Ili, Karzhantau, and Ugam. The distribution area of *J. seravschanica* is constantly decreasing due to intensive logging, forest fires, and excessive cattle grazing. The species has ecological importance in the stabilization of mountain slopes against erosion, for hydrobiological regulation, and as a significant medicinal herb. The species *J. excelsa* M. Bieb., *J. polycarpos* K.Koch (var. *polycarpos* and var. *turcomanica* R.P.Adams), and *J. seravschanica* are morphologically very similar with some difficulties in species identification. For a better understanding of the evolutionary relationship of these species in the *Juniperus* genus, it is important to obtain genetic information on the highly conserved chloroplast (cp) genome. Due to the conserved genomic structure, the cp genome nucleotide sequences are widely used in species distinguishing and reconstructing phylogenetic relationships. Unfortunately, there are no publicly available nucleotide sequences of cp genomes data for *J. polycarpos* (var. *polycarpos* and var. *turcomanica*), *J. excelsa* and *J. seravschanica*. We report the *de novo* assembly of the *J. seravschanica* chloroplast genome by applying next-generation sequencing technology based on Illumina NovaSeq 6000. The assembled cp genome of *J. seravschanica* is 127,609 bp in length and contained 118 genes, including 82 protein-coding genes, 32 transfer RNA genes, and 4 ribosomal RNA genes. In total 152 simple sequence repeats were identified in the chloroplast genome sequence of *J. seravschanica*. The Bioproject (PRJNA883033), Sequence Read Archive (SRR21673293), and GenBank (OL684343) data were deposited at National Center for Biotechnology Information.

**Specifications Table**

**Table:** 

Subject	Omics: Genomics
Specific subject area	Genomics, Forest ecosystem, Environmental science
Type of data	Tables, Figure
How the data were acquired	The data were acquired using the Illumina NovaSeq 6000 (San Diego, USA) sequencer and assembled with SPAdes v. 3.13.0
Data format	Raw data (fastq) and analyzed data (fasta)
Description of data collection	The fresh leaves of *J. seravschanica* were collected from the Turkistan region of Southern Kazakhstan and desiccated in silica gel. Total DNA was isolated from the leaves using the CTAB protocol [1]. The concentration and quality of the extracted DNA were checked by gel electrophoresis in agarose and Nanodrop 2000 spectrophotometry (Thermo Fisher Scientific, Wilmington, DE, USA). Paired-end sequencing was performed using NovaSeq 6000 platform (Illumina, San Diego, CA, USA).
Data source location	• *Institution:* Institute of Plant Biology and Biotechnology • *City/Town/Region:* Almaty • *Country:* Kazakhstan GPS coordinates for collected sample: 42.331250 N, 70.372583 E, altitude 1605 m.
Data accessibility	Repository name: National Center for Biotechnology Information Raw data are available in the Sequence Read Archive (SRA) under BioProject PRJNA883033 with SRA number SRR21673293. The complete chloroplast genome is available under accession number OL684343 Direct URL to data: https://www.ncbi.nlm.nih.gov/sra/PRJNA883033 (SRA) https://www.ncbi.nlm.nih.gov/nuccore/OL684343 (Nucleotide)

## Value of the Data

• The newly sequenced chloroplast genome data of *J. seravschanica* can be useful in plant molecular identification and evaluating phylogenetic relationships at the *Juniperus* genus level.
• Researchers in molecular botany, genomics, and bioinformatics will benefit from these data.
• The detected simple sequence repeats can be used in the development of potentially useful molecular markers and evaluation of genetic diversity in *J. seravschanica* populations and closely related species.

## 1 Objective

Chloroplast genome data can be used in species distinguishing and reconstructing plant evolutionary relationships due to the highly conserved genome structure. There are some difficulties in species identification for morphologically very similar *Juniperus* species *J. excelsa, J. polycarpos* (var. *polycarpos* and var. *turcomanica*), and *J. seravschanica*. Unfortunately, presently there are no publicly available nucleotide sequences of cp genomes data for these listed species. In the present study, we report *de novo* assembled data of the *J. seravschanica* cp genome by applying next-generation sequencing technology based on Illumina NovaSeq 6000. The genome assembly details and annotation for the *J. seravschanica* cp genome were described. The obtained data will provide valuable resources for plant molecular identification and evaluation of phylogenetic relationships at the genus level.

## 2 Data Description

**Fig. 1:**
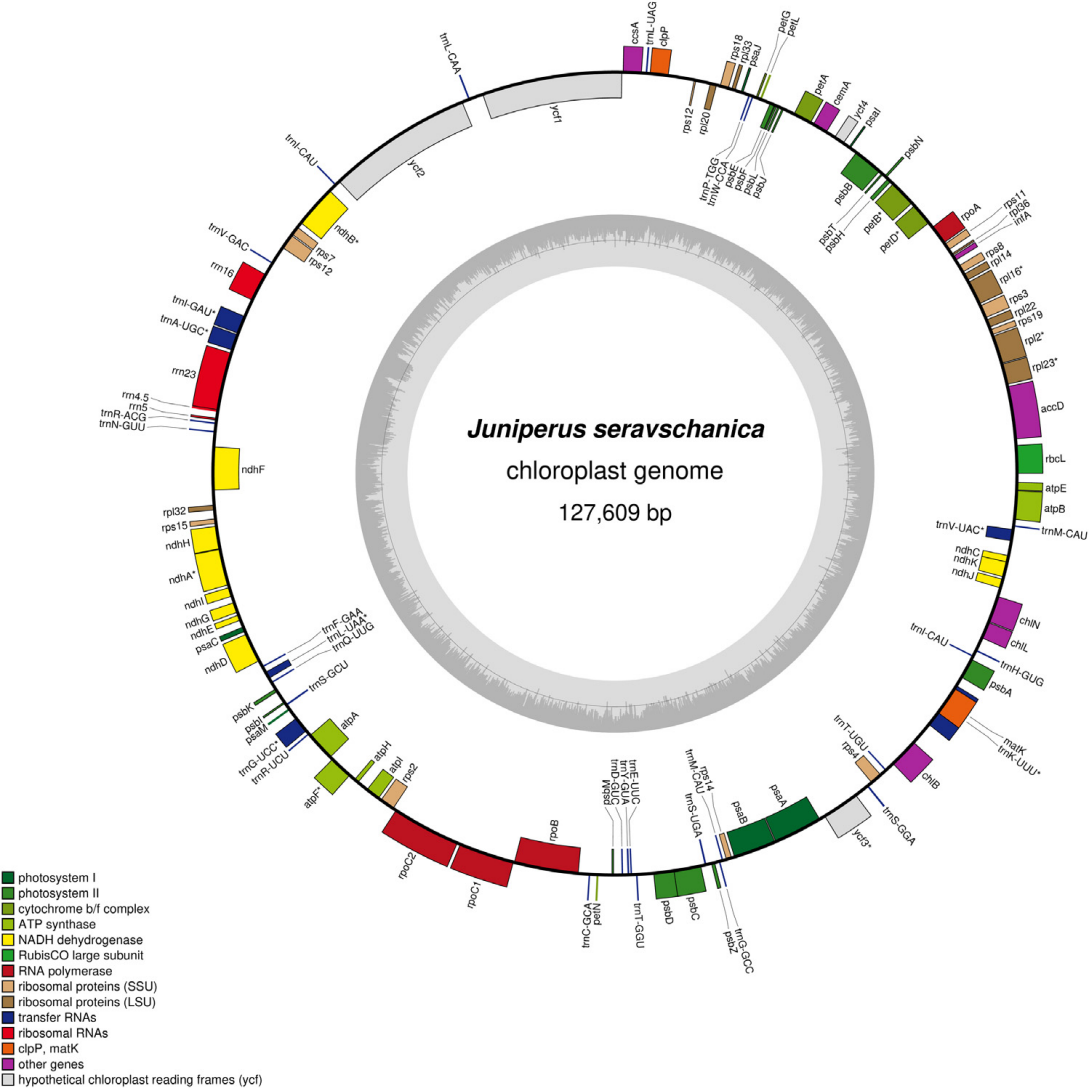
Gene map of the *J. seravschanica* chloroplast genome.

Complete chloroplast genome sequencing using Illumina NovaSeq 6000 of *J. seravschanica* generated about 4 GB of raw data which consisting 24,772,052 paired-end reads with GC content of 34,45% and phred score of 94,39% (Q30) and 98,85% (Q20). The assembled chloroplast genome size of the *J. seravschanica* was 127,609 bp. The structure of the chloroplast genome is circular with a small single-copy region (SSC) and a large single-copy region (LSC). Fig. 1 presented a circular gene map of *J. seravschanica* chloroplast genome.

**Fig. 2:**
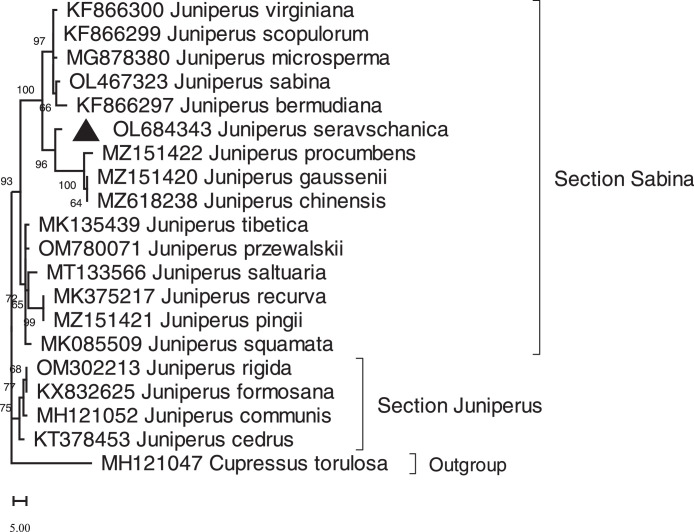
Maximum parsimony concatenated phylogenetic tree based on *mat*K and *rbc*L nucleotide sequences with 1000 bootstraps. ▲ denotes species analyzed in this study.

Maximum parsimony concatenated phylogenetic tree based on *mat*K and *rbc*L nucleotide sequences is given in Fig. 2. The phylogenetic tree separated Juniperus species into two clades which corresponding to the sections *Juniperus* and *Sabina*.

The Bioproject (PRJNA883033), Sequence Read Archive (SRR21673293), and GenBank (OL684343) data were deposited at National Center for Biotechnology Information.The chloroplast genome of *J. seravschanica* encoded 118 genes, including 82 protein-coding genes, 32 transfer RNA (tRNA) genes, and 4 ribosomal RNA (rRNA) genes (Table 1).

**Table 1:** List of genes identified in the *J. seravschanica* cp genome.

Category	Group of genes	Name of genes
Self-replication	Transfer RNA	trnA-UGC*, trnC-GCA, trnD-GUC, trnE-UUC, trnF-GAA, trnG-GCC, trnG-UCC*, trnH-GUG, trnI-CAU (x2), trnI-GAU*, trnK-UUU*, trnL-CAA, trnL-UAA*, trnL-UAG, trnM-CAU (x2), trnN-GUU, trnP-UGG, trnQ-UUG (x2), trnR-ACG, trnR-UCU, trnS-GCU, trnS-GGA, trnS-UGA, trnT-GGU, trnT-UGU, trnV-GAC, trnV-UAC*, trnW-CCA, trnY-GUA
	Ribosomal RNA	*rrn16, rrn23, rrn5, rrn4.5*
	Small subunit of ribosome	rps2, rps3, rps4, rps7, rps8, rps11, rps12*, rps14, rps15, rps18, rps19
	Large subunit of ribosome	rpl14, rpl16*, rpl2*, rpl20, rpl22, rpl23*, rpl32, rpl33, rpl36
	DNA-dependent RNA polymerase	rpoA, rpoB, rpoC1*, rpoC2
	Translational initiation factor	*infA*
Genes for photosynthesis	Rubisco	*rbcL*
	Photosystem I	*psaA, psaB, psaC, psaI, psaJ, psaM*
	Photosystem II	*psbA, psbB, psbC, psbD, psbE, psbF, psbH, psbI, psbJ, psbK, psbL, psbM, psbN, psbT, psbZ*
	ATP synthase	atpA, atpB, atpE, atpF*, atpH, atpI
	Subunits of cytochrome	petA, petB*, petD*, petG, petL, petN
	Chlorophyll biosynthesis	*chlB, chlL, chlN*
	NADH dehydrogenase	ndhA*, ndhB*, ndhC, ndhD, ndhE, ndhF, ndhG, ndhH, ndhI, ndhJ, ndhK
Other genes	Maturase	*matK*
	Protease	*clpP*
	Envelope membrane protein	*cemA*
	Subunit of acetyl-CoA	*accD*
	C-type cytochrome synthesis gene	*ccsA*
Genes of unknown function	Conserved open reading frames	ycf1, ycf2, ycf3**, ycf4

*Note:* One or two asterisks indicate one or two intron-containing genes, respectively, (x2) indicates duplicated genes.

Among these 118 genes, three genes (*trnI-CAU, trnM-CAU* and *trnQ-UUG*) are duplicated, 16 genes (*trnA-UGC, trnG-UCC, trnI-GAU, trnK-UUU, trnL-UAA, trnV-UAC, rps12, rpl16, rpl2, rpl23, rpoC1, atpF, petB, petD, ndhA* and *ndhB*) contain one intron and one gene (*ycf3*) contain two introns. The overall GC content of the *J. seravschanica* assembled chloroplast genome was 35.05%.

In total, 152 simple sequence repeats (SSRs) were determined in *J. seravschanica* plastome by MicroSAtellite (MISA) [2]. Four types of SSRs were detected: 108 mononucleotides, 33 dinucleotides, 5 trinucleotides, and 6 tetranucleotides. Types and amounts of identified SSRs are provided in Table 2.

Among these 152 SSR markers (Supplementary Table 1), 77 (50.7%) SSRs were located in intergenic region, 56 (36.8%) in protein-coding genes, 14 (9.2%) in introns, 3 (2%) in rRNA and 2 in tRNA (1.3%) (rrn23 and trnS-UGA, trnS-GCU, respectively). Most of the SSRs identified in *J. seravschanica* chloroplast genome were located in the intergenic and genic regions (87.5%).

**Table 2:** Types and amounts of simple sequence repeats (SSRs) in the *J. seravschanica* chloroplast genome.

SSR type	Repeat Unit	Ammount	Ratio (%)
Mono	A/T	106	98.1
	C/G	2	1.9
Di	AC/GT	5	15.1
	AG/CT	13	39.4
	AT/AT	15	45.5
Tri	AAG/CTT	3	60
	AAT/ATT	2	40
Tetra	AAAC/GTTT	1	16.7
	AAAG/CTTT	2	33.2
	AAGT/ACTT	1	16.7
	ACCT/AGGT	1	16.7
	ATCC/ATGG	1	16.7

## 3 Experimental Design, Materials and Methods

### 3.1 Plant Material and DNA Extraction

In this study, the fresh leaves of *J. seravschanica* were collected from the Turkistan region of Southern Kazakhstan (42.331250N, 70.372583E). Fresh leaves from *J. seravschanica* samples were desiccated in silica gel and stored at room temperature until DNA extraction. Then, the total DNA was isolated from the leaves under highly sterile conditions using the CTAB protocol [1]. The concentration and quality of the extracted DNA were checked by gel electrophoresis in agarose and Nanodrop 2000 spectrophotometry (Thermo Fisher Scientific, Wilmington, DE, USA).

### 3.2 Library Preparation and Sequencing

Library preparation and cp genome sequencing were conducted by Macrogen Inc. (Seoul, Korea). The library was performed with the TruSeq Nano DNA Kit (Illumina, USA). Paired-end sequencing was performed on Illumina NovaSeq 6000 sequencer based on sequencing by synthesis technology. Generated raw read Fastq format files were used for the genome assembly.

### 3.3 Genome Assembly and Annotation

For accurate genome assembly raw data were quality filtered. Reads in which 90% of the bases had a phred score of 20 or higher were used for assembly. After quality filtering, poly-G trimming was performed using fastp 0.19.4 with a quality phred option as 10 and an unqualified percent limit as 50. In order to reduce biases in the analysis, low-quality reads were removed using Trimmomatic [3]. After filtering, the library for *J. seravschanica* included 24,772,052 total reads. Trimmed reads were used for *de novo* assembly by SPAdes 3.13.0 [4] assembler approach. The complete genome contigs were combined into one contig by joining overlapping DNA segments of each contig. After the draft genome was assembled, the locations of protein genes were predicted and their functions were annotated using Prokka [5]. The circular map (Fig. 1) of the *J. seravschanica* cp genome was generated using Organellar Genome DRAW (OGDRAW) software [6].

### 3.4 Detection of SSR Markers in the Chloroplast Genome

Simple sequence repeats (SSRs) were determined by MISA software [2] with the following thresholds: eight for mononucleotide repeats, four for dinucleotide repeats, four for trinucleotide repeats, three for tetranucleotide repeats, three for pentanucleotide repeats, and three for hexanucleotide repeats. A total of 152 putative SSR markers were identified in the chloroplast genome sequence of *J. seravschanica* (Table 2).

## Ethics Statements

The manuscript adheres to Ethical requirements for publication. The work does not involve studies with animals and humans.

## CRediT authorship contribution statement

**Moldir Yermagambetova:** Methodology, Investigation, Data curation. **Saule Abugalieva:** Investigation, Writing – review & editing. **Yerlan Turuspekov:** Conceptualization, Investigation, Writing – review & editing. **Shyryn Almerekova:** Supervision, Conceptualization, Investigation, Software, Writing – original draft.

## Declaration of Competing Interest

The authors declare that they have no known competing financial interests or personal relationships that could have appeared to influence the work reported in this paper.

## Data Availability

Sequence Read Archive (Original data) (National Center for Biotechnology Information). Complete chloroplast genome of Juniperus seravschanica (Original data) (National Center for Biotechnology Information).

## Appendix Supplementary materials

Image, application 1
