# Supplementary material for: Illumina sequencing data of the complete chloroplast genome of rare species Juniperus seravschanica (Cupressaceae) from Kazakhstan
Source: Data Brief. 2022 Dec 29;46:108866. doi: 10.1016/j.dib.2022.108866 (PMC9850033; doi:10.1016/j.dib.2022.108866)
Supplement: Supplementary file 1 [file mmc1.docx]

**Supplementary Table 1.** Distribution, length and location of simple sequence repeat in the *Juniperus seravschanica* chloroplast sequence

| **cpSSR ID** | **Repeat Motif** | **Length (bp)** | **Start** | **End** | **Annotation** | **Location** |
| --- | --- | --- | --- | --- | --- | --- |
| 1 | (TA)4 | 8 | 1917 | 1924 | accD | Genic |
| 2 | (T)9 | 9 | 2491 | 2499 | accD | Genic |
| 3 | (T)8 | 8 | 3546 | 3553 | accD | Genic |
| 4 | (A)9 | 9 | 5131 | 5139 | rpl23 | Intron |
| 5 | (A)14 | 14 | 7436 | 7449 | rps19 | Genic |
| 6 | (A)9 | 6 | 7474 | 7479 | rps19/rpl22 | Intergenic |
| 7 | (AC)7 | 13 | 7479 | 7492 | rps19/rpl22 | Intergenic |
| 8 | (AG)4 | 8 | 7498 | 7505 | rps19/rpl22 | Intergenic |
| 9 | (AT)5 | 10 | 8313 | 8322 | rps3 | Genic |
| 10 | (A)8 | 8 | 8346 | 8353 | rps3 | Genic |
| 11 | (A)11 | 11 | 10719 | 10729 | rps8 | Genic |
| 12 | (A)9 | 9 | 11021 | 11029 | rps8 | Genic |
| 13 | (A)8 | 8 | 11190 | 11197 | rps8/infA | Intergenic |
| 14 | (A)9 | 9 | 11269 | 11277 | rps8/infA | Intergenic |
| 15 | (T)19 | 19 | 11852 | 11870 | rpl36/rps11 | Intergenic |
| 16 | (A)10 | 10 | 15755 | 15764 | petB | Genic |
| 17 | (A)9 | 9 | 15848 | 15856 | petB | Genic |
| 18 | (T)10 | 10 | 16492 | 16501 | petB/psbH | Intergenic |
| 19 | (AT)4 | 8 | 16814 | 16821 | psbH/psbN | Intergenic |
| 20 | (A)8 | 8 | 19163 | 19170 | psbB/psaI | Intergenic |
| 21 | (CT)4 | 8 | 19210 | 19217 | psaI | Genic |
| 22 | (T)10 | 10 | 19681 | 19690 | ycf4 | Genic |
| 23 | (A)8 | 8 | 20875 | 20882 | cemA | Genic |
| 24 | (A)8 | 8 | 22895 | 22902 | petA/psbJ | Intergenic |
| 25 | (A)11 | 11 | 23128 | 23138 | psbJ | Genic |
| 26 | (A)9 | 9 | 24514 | 24522 | petL/petG | Intergenic |
| 27 | (A)8 | 8 | 24737 | 24744 | petG/trnW-CCA | Intergenic |
| 28 | (AC)5 | 10 | 24745 | 24754 | petG/trnW-CCA | Intergenic |
| 29 | (A)11 | 11 | 25202 | 25212 | trnP-TGG/psaJ | Intergenic |
| 30 | (T)8 | 8 | 25684 | 25691 | psaJ/rpl33 | Intergenic |
| 31 | (A)8 | 8 | 25804 | 25811 | rpl33 | Genic |
| 32 | (AT)5 | 10 | 25958 | 25967 | rpl33/rps18 | Intergenic |
| 33 | (CTTT)3 | 12 | 26502 | 26513 | rps18 | Genic |
| 34 | (A)9 | 9 | 28199 | 28207 | rpl20/clpP | Intergenic |
| 35 | (T)9 | 9 | 28477 | 28485 | rpl20/clpP | Intergenic |
| 36 | (AAT)4 | 12 | 28546 | 28557 | rpl20/clpP | Intergenic |
| 37 | (A)8 | 31 | 28624 | 28631 | rpl20/clpP | Intergenic |
| 38 | (TAAG)3 | 12 | 28760 | 28771 | rpl20/clpP | Intergenic |
| 39 | (AGA)4 | 12 | 28897 | 28908 | rpl20/clpP | Intergenic |
| 40 | (A)8 | 8 | 29157 | 29164 | rpl20/clpP | Intergenic |
| 41 | (A)8 | 8 | 31131 | 31138 | ccsA | Genic |
| 42 | (T)8 | 8 | 32513 | 32520 | ycf1 | Genic |
| 43 | (T)11 | 11 | 33490 | 33500 | ycf1 | Genic |
| 44 | (T)8 | 8 | 33987 | 33994 | ycf1 | Genic |
| 45 | (T)8 | 8 | 34052 | 34059 | ycf1 | Genic |
| 46 | (T)8 | 8 | 34115 | 34122 | ycf1 | Genic |
| 47 | (TTC)5 | 15 | 34163 | 34177 | ycf1 | Genic |
| 48 | (T)9 | 9 | 34547 | 34555 | ycf1 | Genic |
| 77 | (TAT)4 | 12 | 35025 | 35036 | ycf1 | Genic |
| 49 | (A)8 | 8 | 36718 | 36725 | ycf1 | Genic |
| 50 | (T)9 | 9 | 37377 | 37385 | ycf1 | Genic |
| 51 | (T)8 | 8 | 37471 | 37478 | ycf1 | Genic |
| 52 | (T)9 | 9 | 37924 | 37932 | ycf1 | Genic |
| 53 | (A)8 | 8 | 38126 | 38133 | ycf1 | Genic |
| 54 | (T)8 | 8 | 41582 | 41589 | ycf2 | Genic |
| 55 | (A)8 | 8 | 42427 | 42434 | ycf2 | Genic |
| 56 | (T)8 | 8 | 45945 | 45952 | ycf2 | Genic |
| 57 | (T)8 | 8 | 46326 | 46333 | ycf2 | Genic |
| 58 | (T)8 | 8 | 47868 | 47875 | trnI-CAU/ndhB | Intergenic |
| 59 | (T)9 | 9 | 48778 | 48786 | ndhB | Intron |
| 60 | (TTGT)3 | 12 | 49887 | 49898 | ndhB | Exon |
| 61 | (AC)4 | 8 | 51242 | 51249 | rps7/trnV-GAC | Intergenic |
| 62 | (TA)5 | 10 | 51799 | 51808 | rps7/trnV-GAC | Intergenic |
| 63 | (T)8 | 8 | 52003 | 52010 | rps7/trnV-GAC | Intergenic |
| 64 | (T)8 | 8 | 52331 | 52338 | rps7/trnV-GAC | Intergenic |
| 65 | (T)8 | 8 | 52434 | 52441 | rps7/trnV-GAC | Intergenic |
| 66 | (A)8 | 8 | 53038 | 53045 | trnV-GAC/rrn16 | Intergenic |
| 67 | (T)8 | 8 | 55811 | 55818 | trnI-GAU | Intron |
| 68 | (AGGT)3 | 46 | 59428 | 59439 | rrn23 | rRNA |
| 69 | (CT)4 | 46 | 59466 | 59473 | rrn23 | rRNA |
| 70 | (GA)4 | 8 | 59784 | 59791 | rrn23 | rRNA |
| 71 | (T)10 | 10 | 61754 | 61763 | trnN-GUU/ndhF | Intergenic |
| 72 | (C)8 | 8 | 62512 | 62519 | ndhF | Genic |
| 73 | (A)8 | 8 | 64202 | 64209 | ndhF | Genic |
| 74 | (GAAA)3 | 12 | 64485 | 64496 | ndhF | Genic |
| 75 | (A)9 | 9 | 64566 | 64574 | ndhF | Genic |
| 76 | (AC)4 | 8 | 64927 | 64934 | ndhF/rpl32 | Intergenic |
| 78 | (A)9 | 9 | 65314 | 65322 | ndhF/rpl32 | Intergenic |
| 79 | (AT)6 | 12 | 65324 | 65335 | ndhF/rpl32 | Intergenic |
| 80 | (T)9 | 9 | 66416 | 66424 | rps15/ndhH | Intergenic |
| 81 | (AG)4 | 8 | 66863 | 66870 | ndhH | Genic |
| 82 | (T)8 | 8 | 67473 | 67480 | ndhH | Genic |
| 83 | (T)9 | 9 | 68038 | 68046 | ndhA | Exon |
| 84 | (T)12 | 12 | 68235 | 68246 | ndhA | Intron |
| 85 | (A)10 | 10 | 68756 | 68765 | ndhA | Intron |
| 86 | (T)9 | 9 | 68851 | 68859 | ndhA | Intron |
| 87 | (AT)6 | 12 | 74164 | 74175 | ndhD/trnF-GAA | Intergenic |
| 88 | (T)8 | 8 | 75422 | 75429 | psbK | Genic |
| 89 | (T)10 | 10 | 75532 | 75541 | psbK/psbI | Intergenic |
| 90 | (GA)4 | 8 | 76234 | 76241 | trnS-GCU | tRNA |
| 91 | (T)10 | 10 | 77781 | 77790 | trnG-UCC | Intron |
| 92 | (TC)5 | 10 | 77807 | 77816 | trnG-UCC | Intron |
| 93 | (AT)5 | 10 | 79830 | 79839 | atpA/atpF | Intergenic |
| 94 | (TA)7 | 14 | 79840 | 79853 | atpA/atpF | Intergenic |
| 95 | (A)11 | 11 | 80417 | 80427 | atpF | Intron |
| 96 | (T)9 | 9 | 80839 | 80847 | atpF | Exon |
| 97 | (A)9 | 9 | 81209 | 81217 | atpF/atpH | Intergenic |
| 98 | (G)9 | 9 | 81341 | 81349 | atpF/atpH | Intergenic |
| 99 | (A)10 | 10 | 82974 | 82983 | atpI/rps2 | Intergenic |
| 100 | (T)9 | 9 | 83120 | 83128 | rps2 | Genic |
| 101 | (CT)4 | 8 | 83158 | 83165 | rps2 | Genic |
| 102 | (T)11 | 11 | 83473 | 83483 | rps2 | Genic |
| 103 | (T)8 | 8 | 83836 | 83843 | rps2 | Genic |
| 104 | (T)11 | 11 | 84059 | 84069 | rps2/rpoC1 | Intergenic |
| 105 | (T)10 | 10 | 84683 | 84692 | rps2/rpoC1 | Intergenic |
| 106 | (TA)4 | 8 | 86618 | 86625 | rps2/rpoC1 | Intergenic |
| 107 | (TA)4 | 8 | 86708 | 86715 | rps2/rpoC1 | Intergenic |
| 108 | (T)9 | 9 | 93322 | 93330 | rpoB | Genic |
| 109 | (T)9 | 9 | 93356 | 93364 | rpoB | Genic |
| 110 | (TC)4 | 8 | 93657 | 93664 | rpoB | Genic |
| 111 | (T)12 | 12 | 94730 | 94741 | trnC-GCA/petN | Intergenic |
| 112 | (T)10 | 10 | 94744 | 94753 | trnC-GCA/petN | Intergenic |
| 113 | (T)9 | 9 | 95312 | 95320 | petN/psbM | Intergenic |
| 114 | (A)9 | 9 | 95467 | 95475 | petN/psbM | Intergenic |
| 115 | (T)10 | 10 | 95704 | 95713 | psbM/trnD-GUC | Intergenic |
| 116 | (T)9 | 9 | 95876 | 95884 | psbM/trnD-GUC | Intergenic |
| 117 | (CA)4 | 8 | 95927 | 95934 | psbM/trnD-GUC | Intergenic |
| 118 | (A)8 | 8 | 96151 | 96158 | trnD-GUC/trnY-GUA | Intergenic |
| 119 | (TA)4 | 8 | 97465 | 97472 | trnT-GGU/psbD | Intergenic |
| 120 | (CT)4 | 8 | 99494 | 99501 | psbC | Genic |
| 121 | (TA)4 | 8 | 100190 | 100197 | psbC/trnS-UGA | Intergenic |
| 122 | (T)8 | 8 | 100242 | 100249 | psbC/trnS-UGA | Intergenic |
| 123 | (GA)4 | 8 | 100284 | 100291 | trnS-UGA | tRNA |
| 124 | (TA)5 | 10 | 100426 | 100435 | trnS-UGA/psbZ | Intergenic |
| 125 | (A)8 | 8 | 100720 | 100727 | psbZ | Genic |
| 126 | (A)15 | 15 | 106352 | 106366 | psaA | Genic |
| 127 | (A)8 | 8 | 108619 | 108626 | ycf3 | Exon |
| 128 | (A)8 | 8 | 108939 | 108946 | ycf3/trnS-GGA | Intergenic |
| 129 | (A)8 | 8 | 109420 | 109427 | ycf3/trnS-GGA | Intergenic |
| 130 | (T)10 | 10 | 110275 | 110284 | rps4 | Genic |
| 131 | (GA)4 | 8 | 110475 | 110482 | rps4/trnT-UGU | Intergenic |
| 132 | (A)8 | 8 | 110525 | 110532 | rps4/trnT-UGU | Intergenic |
| 133 | (T)8 | 8 | 112971 | 112978 | chlB/trnK-UUU | Intergenic |
| 134 | (T)8 | 8 | 113103 | 113110 | chlB/trnK-UUU | Intergenic |
| 135 | (T)9 | 9 | 113217 | 113225 | chlB/trnK-UUU | Intergenic |
| 136 | (AGA)6 | 18 | 113440 | 113457 | chlB/trnK-UUU | Intergenic |
| 137 | (T)9 | 9 | 113561 | 113569 | chlB/trnK-UUU | Intergenic |
| 138 | (T)9 | 9 | 113705 | 113713 | chlB/trnK-UUU | Intergenic |
| 139 | (A)9 | 57 | 114016 | 114024 | trnK-UUU | Intron |
| 140 | (T)9 | 57 | 114064 | 114072 | trnK-UUU | Intron |
| 141 | (T)8 | 8 | 114709 | 114716 | trnK-UUU | Intron |
| 142 | (ATGG)3 | 12 | 114996 | 115007 | trnK-UUU | Intron |
| 143 | (T)8 | 8 | 118097 | 118104 | trnI-CAU/trnH-GUG | Intergenic |
| 144 | (A)13 | 13 | 118137 | 118149 | trnI-CAU/trnH-GUG | Intergenic |
| 145 | (A)11 | 11 | 118391 | 118401 | trnH-GUG/chlL | Intergenic |
| 146 | (A)9 | 9 | 118538 | 118546 | trnH-GUG/chlL | Intergenic |
| 147 | (A)8 | 8 | 118887 | 118894 | trnH-GUG/chlL | Intergenic |
| 148 | (AT)4 | 8 | 121603 | 121610 | chlN/ndhJ | Intergenic |
| 149 | (T)9 | 9 | 121718 | 121726 | chlN/ndhJ | Intergenic |
| 150 | (A)9 | 9 | 123617 | 123625 | ndhC/trnV-UAC | Intergenic |
| 151 | (TC)4 | 8 | 124470 | 124477 | trnV-UAC | Intron |
| 152 | (A)12 | 12 | 126753 | 126764 | atpB/atpE | Intergenic |
